# The Alumni Association Prize of the College of Physicians and Surgeons of New York

**Published:** 1885-07

**Authors:** 


### NEWS ITEMS.

THE ALUMNI ASSOCIATION PRIZE OF THE COLLEGE OF PHYSICIANS AND SURGEONS, N. Y.—This prize of five hundred dollars, offered to the competition of the Alumni of the College, will be awarded at the next Annual Commencement, May 11, 1866.

The competing Essays must embody the result of *original research* upon some medical subject. They must bear a motto or device, and be accompanied by a sealed envelope bearing the same motto, and containing the author's name and address.

Essays should be sent before April 1, 1886, to any of the committee undersigned in New York city.

E. C. SEGUIN, M. D.
J. WEST ROOSEVELT, M. D.
M. ALLEN STARR, M. D.

